# ﻿Typification of the name *Arthopyreniaparolinii* Beltr. (Ascomycota, Dothideomycetes, Pleosporales, Arthopyreniaceae)

**DOI:** 10.3897/mycokeys.104.109420

**Published:** 2024-04-16

**Authors:** Deborah Isocrono, Sonia Ravera

**Affiliations:** 1 Department of Agricultural, Forest and Food Sciences (DISAFA), University of Torino, Largo Paolo Braccini 2, Torino, 10095 Grugliasco, Italy University of Torino Torino Italy; 2 Department of Biological, Chemical and Pharmaceutical Sciences and Technologies (STeBiCeF), University of Palermo, via Archirafi 38, 90123 Palermo, Italy University of Palermo Palermo Italy

**Keywords:** A.B. Massalongo Herbarium, Beltramini, lectotype, lichen, nomenclature

## Abstract

*Arthopyreniaparolinii* Beltr. is one of the few species of the lichen genus *Arthopyrenia* A. Massal. described by Italian authors of the XIX century, lacking type formal association. In this regard, the name *Arthopyreniaparolinii* is hereby lectotypified using a specimen stored in the lichen herbarium of A.B. Massalongo at VER. Additional original material was found only at M, while another specimen at MSNVE, labelled as *Spermatodiumparolinii*, although referable to this species, should not be considered as original material. *Arthopyreniaparolinii* is among the least well-known species in the genus. Given the genus *Arthopyrenia* is still very poorly known, it is important to clarify the original material of the species and propose the lectotyping. The selected lectotype is the only sample among the analyzed ones reporting complete data on the locus classicus; it conforms to the characters described in the protologue and comes from the Herbarium Beltramini.

## ﻿Introduction

The lichen genera *Arthopyrenia* A. Massal. and *Naetrocymbe* Körb. both include poorly understood non-lichenized and lichenized fungi (Isocrono et al. 2021). Despite their wide distribution, species belonging to these genera are often overlooked and pose taxonomic challenges (Hongsanan et al. 2020; Thiyagaraja et al. 2021).

As part of the research carried out by the authors focusing on the occurrence of *Arthopyrenia* and *Naetrocymbe* in Italy (Ravera 2006, 2014; Puntillo and Ravera 2013; Ravera and Isocrono 2021; Isocrono and Ravera 2022) we aimed to establish the identity of *Arthopyreniaparolinii* Beltr., a neglected species not yet typified. *A.parolinii*, such as several other multiseptated species described by Italian authors of the XIX Century –e.g., *A.cembricola* (Anzi) Lettau, *A.cinerescens* A. Massal., *A.molinii* Beltr., etc.– still await a critical study. Given the challenges relating to the genus *Arthopyrenia* which according to Wijayawardene et al. (2022) includes 5 + approximately 100 orphaned species - i.e. species that have been named and formally described, but have not been updated and reassessed following a revision of the genus - it is crucial to analyze and clarify the poorly known original material of such species and propose the lectotyping.

We have therefore examined several historical lichen samples probably attributable to *Arthopyreniaparolinii* Beltr. Among the checked exsiccates, the sample of *A.parolinii* stored in the Abramo Bartolomeo Massalongo lichen herbarium in VER, revealed that it originates from the Francesco Beltramini de’ Casati collection and it was collected in the location reported in the protologue, i.e. locus classicus.

Francesco Beltramini de’ Casati’s (1828–1903) primary botanical interest lay in lichens. In this field, he published a richly illustrated work on the lichen flora of Bassano del Grappa, Vicenza, Italy (Beltramini de’ Casati 1858). Massalongo, his friend and mentor, is honored in this work.

The possibility that Beltramini’s lichenological collections may have merged into the renowned lichenologist’s herbarium is plausible because of Massalongo’s well-known friendship with his fellow countryman and student Beltramini.

## ﻿Methods

This study is based on: i) analysis of the protologue, ii) pinpointing the location of the locus classicus, iii) search for the original material, iv) examination of specimens in M (Botanische Staatssammlung München), MSNVE (Lichenotheca Veneta by Vittore Trevisan kept at Natural History Museum of Venice Giancarlo Ligabue), and VER (Herbarium of A.B. Massalongo at the Civic Natural History Museum of Verona). High-resolution digital images from MSNVE and M were also consulted.

Macroscopic and microscopic characteristics were observed in dried specimens with a Zeiss dissecting microscope equipped with a Leica camera. Microscopic characters were examined from hand-cut sections and squashes mounted in a 5% KOH solution from dried specimens with a Zeiss Axioscope optical microscope equipped with AxioCam MRc camera (Zeiss, Welwyn Garden City, UK).

Typification follows the International Code of Nomenclature for algae, fungi and plants (Turland et al. 2018).

## ﻿Results and discussion

### ﻿Original material of *Arthopyreniaparolinii*

In the protologue of *Arthopyreniaparolinii*, Beltramini provides a diagnosis, a more detailed description, and some drawings showing perithecium, asci, and multiseptated spores. Particularly, the description reports:

"*Thallo primum hypophlaeodico, tandem epiphlaeodico, arachnoideo effuso, cinereo-fusco. Apotheciis minutis, creberrimis, subemerso-sessilibus, hemisphaerico-conoideis, atris. Ascis clavato-ventricosis, apice rotundato-truncatis, basi in petiolum rudem attenuatis, octosporis, absque paraphysibus; sporidiis ovoideo-elongatis, basi subclavatis, 6–8-locularibus, diametro quadruplo vel quintuplo longioribus*" (Beltramini de’ Casati 1858).

According to Beltramini’s explanation in the paper, the epithet is in honor of the “Cavaliere Nob. Alberto Parolini” (1788–1867) a renowned Italian naturalist known for his rich botanical garden named Parolini Garden at Bassano del Grappa, and for his donation of a sizable collection of natural history objects to the local civic museum.

The collecting site of the sample is mentioned as “le tiglie nel passeggio di Belvedere in Bassano” [on Tilia along Belvedere stroll in Bassano].

**Figure 1.**
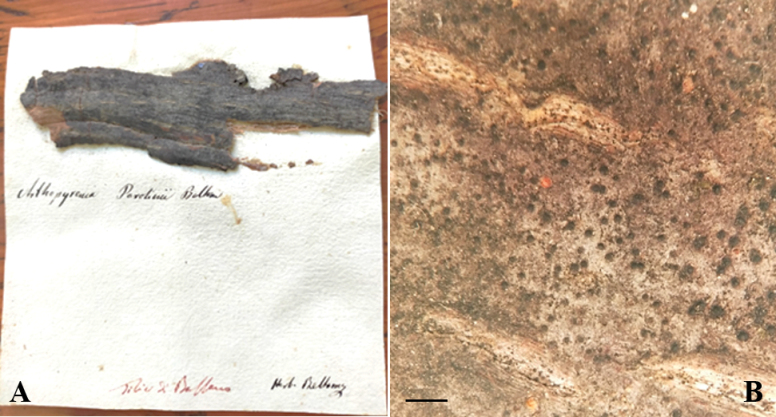
Exsiccata of *Arthopyreniaparolinii* Beltramini from the lichen herbarium of A.B. Massalongo in VER**A** fragment of linden bark, colonized by the lichen and glued to the herbarium sheet with the name of the species and note “Herb. Beltramini” written by Massalongo in black ink, and the locality of collection, in red ink, written by Beltramini **B** detail of thallus and perithecia in surface view. Scale bar: 1 mm.

The sample stored in the Lichenological Herbarium of A.B. Massalongo in Verona (Fig. 1A, B) is made up of a piece of bark glued to the herbarium sheet. The label reports the location “Tilia di Bassano” [Tilia of Bassano del Grappa] and the note “Herb. Beltramini”. The two sentences display different handwriting: a calligraphic comparison made with original material preserved in the Library of University of Padua (see https://phaidra.cab.unipd.it/) allowed us to identify the locality, in red ink, as written by Beltramini while the name of the species and the herbarium attribution as written by Massalongo.

We also found a second undated collection of *Arthopyreniaparolinii* in Staatliche Naturwissenschaftliche Sammlungen Bayerns Herbarium (M). This sample –M-0207340– shows the same set-up and comes from Ferdinand Christian Gustav Arnold personal herbarium.

**Figure 2.**
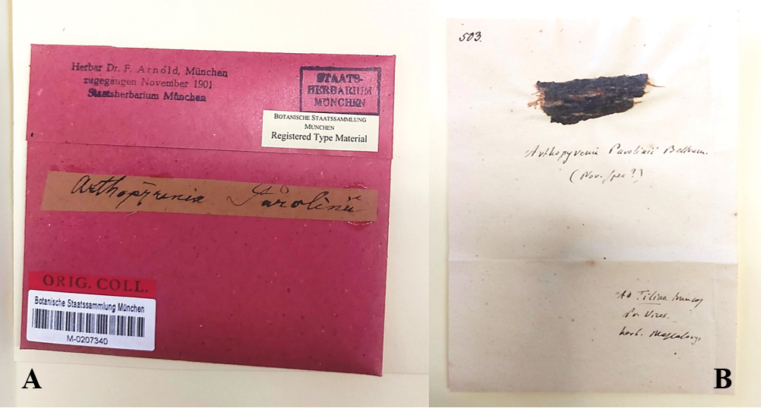
Exsiccata of *Arthopyreniaparolinii* Beltramini from M Lichen herbarium (M-0207340) registered as “Type Material” **A** the original label from Arnold personal herbarium **B** a fragment of linden bark, colonized by the lichen, glued to the herbarium sheet with the name of the species accredited to Beltramini and a few notes written by Massalongo.

On the sheet (Fig. 2), some notes are reported: the name of the species accredited to Beltramini, the note “nov. spec ?”, the substratum “ad Tiliae truncos”, the location “Pr Vicez.” [Province of Vicenza], the herbarium from which the sample was taken “herb. Massalongo”. All these notes appear to be written by Massalongo.

Consequently, among the few original materials that currently exist, we designate the specimen stored in VER as lectotype, as this is the most complete, and informative, and in line with the protologue.

### ﻿Description of the lectotype

**Figure 3.**
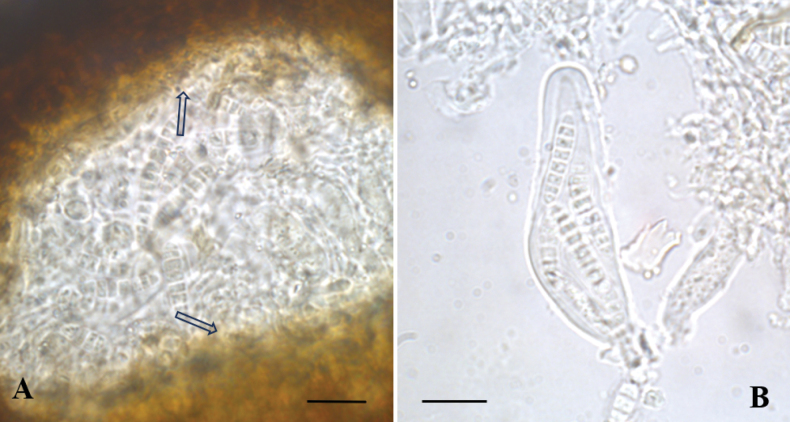
*Arthopyreniaparolinii***A** vertical section through a perithecium, arrows showing hamathecium and K- excipulum **B** bitunicate asci and pluriseptate ascospores in 10% KOH. Scale bars: 15 µm.

**Thallus**: epiphloeodal, dark gray, thin, non-lichenized (Fig. 1B). **Ascomata**: perithecial 0.15–0.2 mm, black, subglobose, ± circular, numerous, scattered, superficial, many with depressed ostiole; ascomatal wall of textura intricata, black, not continuous below the hamathecium; involucrellum reddish brown, clypeate, amorphous pigment localized in the cell wall; excipulum colourless, scarcely discernible; the wall pigment remains brown in K (Fig. 3A, B). **Hamathecium**: moniliform pseudoparaphyses dissolving and leaving only fragments embedded in gel; periphysoids not present; asci: 60–70 × 24–27 µm, cylindric-clavate, bitunicate with a distinct apical region lacking a nasse, dehiscence typically fissitunicate. **Ascospores**: 21–22 × 4–5 µm, 8 per ascus, irregularly arranged, colourless, clavate with rounded apices, 5–7-septate, slightly constricted at the septum; perispore indistinct. **Pycnidia**: not observed. **Chemistry**: spot tests negative.

### ﻿Notes

Studies on *Arthopyrenia* species with 5–7 septate spores in Europe are still lacking. Several species formally named and described by Beltramini (e.g. *Arthopyreniamolinii* Beltr.) (Isocrono and Ravera 2022) and Massalongo have not been updated or re-evaluated in recent studies. The main reason is the difficulty of working with material dating back to the 19^th^ century, for which there are an extremely limited number of samples, which are often difficult to locate and obtain in loan, and in many cases these species are only known from the locus classicus (Nimis et al. 2018).

Among current and known species, *Arthopyreniagrisea* (Schaer.) Körb. and *Arthopyreniaplatypyrenia* (Nyl.) Arnold are the most similar to *A.parolinii*.

According to Foucard (1992)*A.grisea* seems to be the most proper name for some varieties of *A.personii* Massal. with moniliform pseudoparaphyses, obpyriform asci and (3-)5(-6) septate clavate spores. The main distinction between *A.parolinii* and the varieties of *A.personii* synonymized with *A.grisea* (see e.g. Nimis 2023) is that *A.parolinii* permanently shows 5–7 septate spores. For its part, *A.platypyrenia*, is a rare but well-known species, usually collected on *Hederahelix* L. (Coppins and Orange 2009). It is characterized by a broad lateral ostiole and different ascus and spores. Particularly, spores are usually ellipsoidal to fusiform-ellipsoidal, 3- to 4- (to 7-) septate, constricted at all the septa, with a gelatinous sheath 2–3 µm thick and larger (24–30 × 8–10) than the ones of *A.parolinii*.

### ﻿The locus classicus

**Figure 4.**
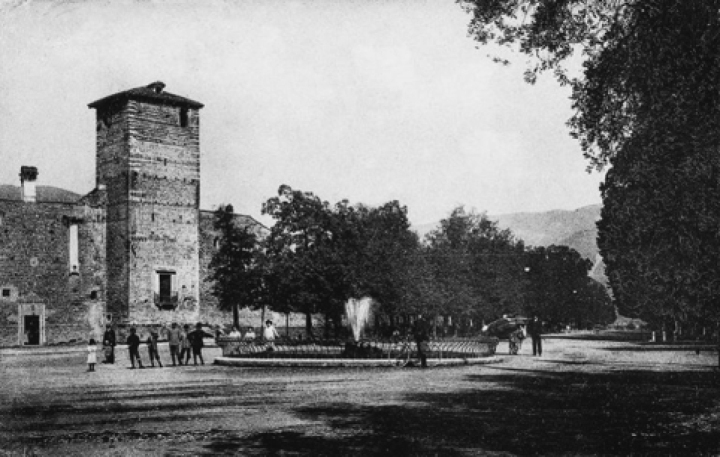
*Arthopyreniaparolinii* Beltr locus classicus (i.e. “Passeggio pubblico di Belvedere o Fosse”) depicted in a 1917 postcard (from: Bordignon 2016). The image shows the original lime trees that are mentioned in the protologue of *A.parolinii* before their removal.

In Bassano del Grappa, an Italian town located in the province of Vicenza, Italy, the avenue once known as Viale Belvedere is now named as Viale delle Fosse. This avenue was built in 1790 when the moats surrounding the Visconti walls were covered. After this work, a double row of linden trees (Fig. 4) and various statues were added to the avenue which, starting from the Porta delle Grazie, reached the Parolini Garden. This new tree-lined avenue was called “Passeggio pubblico di Belvedere o Fosse” [Public Walk of Belvedere or Fosse], the same name reported in the *A.parolinii* protologue.

220 of the original linden trees were removed for plant health reasons during World War I, and they were replaced by American elms, effectively preventing us from searching for current material on the original phorophytes (Bordignon 2016).

### ﻿Samples of *Arthopyreniaparolinii*

Vittore Benedetto Antonio Trevisan transferred the epithet to *Spermatodium* Feé (Trevisan de St-Léon 1860) on the basis of the spores’ characteristics. In 1869 Trevisan issued eight fascicles of his Lichenotheca Veneta. A single specimen of *Spermatodiumparolinii* is included among these exsiccates deposited at MSNVE (MSNVE-24815) (see: Lichenotheca Veneta del Conte Vittore Trevisan 2023).

Considering the stormy relations between Trevisan and Massalongo (Nimis and Hawksworth 1994) and the absence of notes on the specimen – unlike other samples from Massalongo in MSNVE – it is conceivable that the collection was carried out by Trevisan himself and in our opinion it should be not original material.

### ﻿Typification

*Arthopyreniaparolinii* Beltr., Lich. Bassan.: 239. 1858 ≡ *Spermatodiumparolinii* (Beltr.) Trevis., Conspect. Verruc.: 11. 1860 ≡ *Santessoniolichenparolinii* (Beltr.) Tomas. & Cif., Arc. Bot. Ital.: 5. 1952 ≡ *Giacominiaparolinii* Cif. & Tomas., Atti Ist. Bot. E Lab. Critt. Univ. Pavia: 256. 1954 – **Lectotype** (designated here): “*Arthopyreniaparolinii*”, Herb. A.B. Massalongo (VER!). MycoBank No: 10015170

## ﻿Additional information

### Author ORCIDs

Deborah Isocrono https://orcid.org/0000-0003-0570-0674

Sonia Ravera https://orcid.org/0000-0001-5223-7964

### Data availability

All of the data that support the findings of this study are available in the main text.
